# Expression of ID4 protein in breast cancer cells induces reprogramming of tumour-associated macrophages

**DOI:** 10.1186/s13058-018-0990-2

**Published:** 2018-06-19

**Authors:** Sara Donzelli, Elisa Milano, Magdalena Pruszko, Andrea Sacconi, Silvia Masciarelli, Ilaria Iosue, Elisa Melucci, Enzo Gallo, Irene Terrenato, Marcella Mottolese, Maciej Zylicz, Alicja Zylicz, Francesco Fazi, Giovanni Blandino, Giulia Fontemaggi

**Affiliations:** 1 Oncogenomics and Epigenetics Unit, IRCCS Regina Elena National Cancer Institute, Via Elio Chianesi 53, 00144 Rome, Italy; 2 Department of Molecular Biology, International Institute of Molecular and Cell Biology in Warsaw, Księcia Trojdena 4, 02-109 Warsaw, Poland; 3 Department of Anatomical, Histological, Forensic & Orthopaedic Sciences, Section of Histology & Medical Embryology, Sapienza University of Rome, Via A. Scarpa, 16, 00161 Rome, Italy; 4 Laboratory affiliated with Istituto Pasteur Italia-Fondazione Cenci Bolognetti, Rome, Italy; 5 Pathology Department, IRCCS Regina Elena National Cancer Institute, Via Elio Chianesi 53, 00144 Rome, Italy; 6 Biostatistics Unit, Scientific Direction, IRCCS Regina Elena National Cancer Institute, Via Elio Chianesi 53, 00144 Rome, Italy

**Keywords:** ID4, Breast cancer, TAMs, miR-107, HIF-1A, GRN

## Abstract

**Background:**

As crucial regulators of the immune response against pathogens, macrophages have been extensively shown also to be important players in several diseases, including cancer. Specifically, breast cancer macrophages tightly control the angiogenic switch and progression to malignancy. ID4, a member of the ID (inhibitors of differentiation) family of proteins, is associated with a stem-like phenotype and poor prognosis in basal-like breast cancer. Moreover, ID4 favours angiogenesis by enhancing the expression of pro-angiogenic cytokines interleukin-8, CXCL1 and vascular endothelial growth factor. In the present study, we investigated whether ID4 protein exerts its pro-angiogenic function while also modulating the activity of tumour-associated macrophages in breast cancer.

**Methods:**

We performed IHC analysis of ID4 protein and macrophage marker CD68 in a triple-negative breast cancer series. Next, we used cell migration assays to evaluate the effect of ID4 expression modulation in breast cancer cells on the motility of co-cultured macrophages. The analysis of breast cancer gene expression data repositories allowed us to evaluate the ability of ID4 to predict survival in subsets of tumours showing high or low macrophage infiltration. By culturing macrophages in conditioned media obtained from breast cancer cells in which ID4 expression was modulated by overexpression or depletion, we identified changes in the expression of ID4-dependent angiogenesis-related transcripts and microRNAs (miRNAs, miRs) in macrophages by RT-qPCR.

**Results:**

We determined that ID4 and macrophage marker CD68 protein expression were significantly associated in a series of triple-negative breast tumours. Interestingly, ID4 messenger RNA (mRNA) levels robustly predicted survival, specifically in the subset of tumours showing high macrophage infiltration. In vitro and in vivo migration assays demonstrated that expression of ID4 in breast cancer cells stimulates macrophage motility. At the molecular level, ID4 protein expression in breast cancer cells controls, through paracrine signalling, the activation of an angiogenic programme in macrophages. This programme includes both the increase of angiogenesis-related mRNAs and the decrease of members of the anti-angiogenic miR-15b/107 group. Intriguingly, these miRNAs control the expression of the cytokine granulin, whose enhanced expression in macrophages confers increased angiogenic potential.

**Conclusions:**

These results uncover a key role for ID4 in dictating the behaviour of tumour-associated macrophages in breast cancer.

**Electronic supplementary material:**

The online version of this article (10.1186/s13058-018-0990-2) contains supplementary material, which is available to authorized users.

## Background

Breast cancer (BC) is the most common cancer in women worldwide and remains a leading cause of cancer death [1]. It is a heterogeneous disease with multiple subtypes that display different patterns of gene expression, prognosis and response to treatment [2]. Metastasis, which is responsible for over 90% of BC deaths, is regulated to a great extent by reciprocal interactions between cancer cells and immune cells in the tumour microenvironment [3, 4].

Tumour-associated macrophages (TAMs), which are part of the adaptive immune response, constitute a major portion of the leucocyte infiltrate found in breast tumours and tightly control angiogenic switch and progression to malignancy in BC [5]. Tumour cells actively recruit macrophages and educate them to be pro-tumourigenic [6, 7]. TAMs exhibit potent proliferative capacity upon their differentiation from inflammatory monocytes, and the presence of intra-tumoural proliferating macrophages was significantly correlated with high-grade, hormone receptor-negative tumours and a basal-like subtype of BC [7, 8]. The number of proliferating macrophages was also a significant predictor of recurrence and survival [9].

Several reports suggest that TAMs adopt a trophic immunosuppressive phenotype that is functionally reminiscent of the alternatively activated type II (M2) macrophages [10]. However, TAMs present great phenotypic diversity depending on the combinations of stimuli received in the tumour stroma, and it has been proposed that multiple subpopulations of TAMs exist within tumours, which probably change temporally during tumour development and geographically on the basis of their location within the tumour microenvironment [11, 12]. Functionally, TAMs have been shown to facilitate tumour angiogenesis, invasion, intravasation and metastasis in animal models [13, 14] and are now recognised as important therapeutic targets in the treatment of cancer [15].

ID4 is a member of the ID family of proteins (inhibitors of differentiation, ID-1 to ID-4) that act as dominant-negative regulators of basic helix-loop-helix transcription factors [16]. Studies have indicated that ID proteins are associated with loss of differentiation, stemness, unrestricted proliferation, and neoangiogenesis in diverse human cancers. In the context of BC, ID4 is highly expressed in triple-negative breast cancer (TNBC), 70% of which belong to the basal-like breast cancer (BLBC) molecular subtype [17, 18]. Accordingly, ID4 was repeatedly identified as a component of BLBC-associated molecular signatures [19]. Recent evidence suggests an emerging role for ID4 as a lineage-dependent proto-oncogene that is overexpressed and amplified in BLBCs and is associated with stem-like phenotype and poor prognosis in this subtype and in TNBC [17, 20–23].

At the molecular level, ID4 has been shown to be responsible for the downregulation of BRCA1 promoter activity [24], and consequently, ID4 expression is inversely correlated with that of BRCA1 [20, 23, 25, 26]. In addition, clinical data have indicated preferential ID4 amplification in BRCA1 mutant cases [23, 27]. We previously reported that ID4 protein results in induction of chemokine (C-X-C motif) ligand 1 (CXCL1) and interleukin (IL)-8 pro-angiogenic cytokines and in enhanced angiogenic potential of BC [28, 29]. Moreover, mutant p53 proteins transcriptionally induce ID4, and a complex containing ID4 and mutant p53 proteins is responsible for the synthesis of pro-angiogenic vascular endothelial growth factor (VEGF) isoforms in BC [30].

To fully explore the mechanisms through which ID4 controls BC angiogenesis, we investigated whether it was able to modulate TAM activity. We report that ID4 expression in BC cells is indeed able to reprogramme the expression of angiogenesis-related genes in macrophages through a paracrine VEGF-dependent effect. In particular, we observed the ID4-dependent induction of hypoxia-inducible factor (HIF)-1A, whose expression in macrophages suppresses T-cell function and promotes progression in BC [31], and of granulin (GRN), which was previously reported to control macrophage activity in autoimmune diseases [32]. Of note, microRNAs (miRNAs, miRs) of the miR-15b/107 group, which target these angiogenesis-related factors, were concomitantly downregulated. Our data also showed that high ID4 mRNA expression level is associated with reduced distant metastasis-free survival (DMFS) and overall survival (OS), specifically in patients carrying tumours highly infiltrated by macrophages.

## Methods

### Cell cultures and transfections

The SKBR3, MDA-MB-468, HL60 and U937 cell lines were grown at 37 °C with 5% CO_2_ and maintained in RPMI medium containing 10% heat-inactivated FBS and penicillin/streptomycin. HL60 and U937 cells were differentiated by treatment with 1,25-dihydroxyvitamin D_3_ (VitD3) (Sigma-Aldrich, St. Louis, MO, USA) at a concentration of 250 ng/ml. Monocytic differentiation was assessed by fluorescence-activated cell sorting (FACS) as previously reported [33] using allophycocyanin (APC) anti-human CD11b (BD Biosciences, San Jose, CA, USA), PerCP-Cy5.5 (peridinin chlorophyll protein complex-cyanine 5.5) anti-human CD14 (BD Biosciences) and phycoerythrin-immunoglobulin G1 (PE-IgG1) isotype control (eBioscience Inc., San Diego, CA, USA) antibodies for the evaluation of CD11b-CD14 co-expression as a marker of monocytic differentiation. A minimum of 10,000 events were collected for each sample with a flow cytometer (CyAN ADP; Beckman Coulter Life Sciences, Brea, CA, USA) using Summit 4.3 software (Beckman Coulter Life Sciences) for data acquisition and analysis.

An expression vector containing a hemagglutinin (HA)-tagged ID4 coding sequence [28] or control empty vector was transfected in cancer cells using Lipofectamine 2000 reagent (Thermo Fisher Scientific, Waltham, MA, USA) in ID4 overexpression experiments. RNAiMAX reagent (Thermo Fisher Scientific, Waltham, MA, USA) was used to transfect small interfering RNAs (siRNAs) in BC cells. Sequences of siRNAs directed to ID4 were previously reported [30]. Monocytic cell lines were transfected with plasmids, mimic and locked nucleic acid (LNA) oligonucleotides (Dharmacon, Lafayette, CO, USA) using the TransIT-X2® Dynamic Delivery System (Mirus Bio LLC, Madison, WI, USA) following the manufacturer’s instructions. Full-length cDNA (including 5′-UTR and 3′-UTR) of human GRN (NM_002087.2), cloned in the pCMV6-XL5 plasmid vector, was generously provided by Dr. Peter Nelson.

Mouse bone marrow-derived macrophage precursors were obtained from rodents by flushing the femurs and tibias with 2% FBS in PBS. Differentiation was induced by culturing precursors in colony-stimulating factor 1 (CSF1)-rich conditioned media (CM) derived from L929 fibroblast cell culture. Differentiation was evaluated by FACS analysis using the following antibodies: anti-mouse F4/80 antigen APC (17-4801; eBioscience), Ly-6G (Gr-1) APC (17-5931; eBioscience, San Diego, CA, USA), CD14 PE (12-0141; eBioscience, San Diego, CA, USA) and CD107b (Mac-3) PE (12-5989; eBioscience, San Diego, CA, USA).

Human peripheral blood-derived monocytes were isolated from blood donors using Lymphoprep solution (Axis-Shield, Dundee, UK) followed by isolation of CD14^+^ cells with the Monocyte Isolation Kit II (Miltenyi Biotec, Bergisch Gladbach, Germany). Differentiation was achieved through 1-week culturing in RPMI medium containing recombinant CSF1 (human macrophage colony-stimulating factor, catalogue number 8929SC; Cell Signaling Technology, Danvers, MA, USA).

CM from BC cells were prepared by culturing cells for 24 hours in serum-free RPMI medium. CM were centrifuged to eliminate cell residues before preparation of aliquots and storage at − 80 °C. When si-ID4 BC cells were used to prepare CM, we always collected CM before 48 hours from transfection because of proliferation of cells being delayed after this time point in the si-ID4 condition (Additional file 1: Figure S3).

### In vitro and in vivo macrophage migration assays

Migration of mouse bone marrow-derived macrophages in response to SKBR3 cells was evaluated using 3-μm-pore Boyden chambers (Corning Inc., Corning, NY, USA). Infiltration of F4/80^+^ macrophages in Matrigel plugs containing CM from BC MDA-MB-468 cells was evaluated by subcutaneous inoculation of a solution composed of 500 μl of Matrigel (BD Biosciences) and 50 μl of a 10 × concentration of CM. In the negative control, the CM was replaced with serum-free medium. Plugs were recovered at day 7, fixed for 18–24 hours in 4% (vol/vol) buffered formaldehyde, and then processed with paraffin wax. IHC was performed using F4/80 antibody (MA5-16363; Pierce Biotechnology, Rockford, IL, USA). All procedures involving animals and their care were conducted in conformity with institutional guidelines, which are in compliance with national and international standards.

### IHC

Tumours from 62 patients included in this study were previously described in a study by Novelli et al. [34], which was reviewed and approved by the ethics committee of the Regina Elena National Cancer Institute and contained data for which written informed consent was obtained from all patients. Characteristics of these patients are included in Additional file 2: Table S1. BC specimens for IHC analysis were fixed for 18–24 hours in 4% (vol/vol) buffered formaldehyde and then processed with paraffin wax. Anti-ID4 (MAB4393; EMD Millipore, Billerica, MA, USA), anti-oestrogen receptor (clone 6F11; Novocastra, Florence, Italy), anti-progesterone receptor (anti-PgR, clone 1A6; Novocastra), and anti-HER2 (A0485; Dako, Milan, Italy) were evaluated by IHC in 5-μm-thick paraffin-embedded tissues. Monoclonal antibodies (mAb) directed against ID4 were incubated at a dilution of 1:200 overnight at 4 °C, and anti-ER and anti-PgR mAb and the polyclonal antibody anti-HER2 were incubated for 60 minutes at room temperature. Immunoreactions were revealed by a streptavidin-biotin enhanced immunoperoxidase technique (Super Sensitive MultiLink; BioGenex, Fremont, CA, USA) in an autostainer (Bond III; Leica Biosystems, Wetzlar, Germany). Diaminobenzidine (DAB) was used as a chromogenic substrate. Evaluation of the IHC data was performed independently and in a blinded manner by two investigators (EG and EM).

### Immunocytochemistry and immunofluorescence

For immunocytochemistry assay, cells were seeded onto glass coverslips (Paul Marienfeld, Lauda-Königshofen, Germany) in 6-well dishes (Corning Inc.) at 4 × 10^4^ cells/well, cultured with RPMI or CM, and fixed with 4% formaldehyde in PBS for 15 minutes at room temperature. Cells were permeabilized with 0.25% Triton X-100 in PBS for 10 minutes. After washing with PBS, the coverslips were incubated with anti-ID4 antibody diluted in 5% bovine serum albumin (BSA)/PBS for 2 hours at room temperature. Cells were incubated with peroxidase inhibitor before primary antibody incubation. Protein staining was revealed through DAB enzymatic reaction, and nuclei were counterstained with haematoxylin.

For immunofluorescence, cells grown in the presence of RPMI or CM (48 hours), as well as cells transfected with mimic oligonucleotides (48 hours), were concentrated onto microscope slides using cytospin and fixed and permeabilized as already described. Slides were blocked for 30 minutes in 5% BSA/PBS at room temperature and then incubated with an anti-HIF-1A antibody (A300-286A; Bethyl Laboratories, Montgomery, TX, USA) diluted in 5% BSA/PBS for 2 hours at room temperature. Cells were incubated with secondary antibody Alexa Fluor 594 (1:500; Thermo Fisher Scientific) for 45 minutes. Nuclei were stained with DAPI (Thermo Fisher Scientific).

### Western blotting and antibodies

For the Western blot analysis, cells were lysed in radioimmunoprecipitation assay buffer or 8 M urea. The protein concentration was measured using a Bio-Rad protein assay kit (Bio-Rad Laboratories, Hercules, CA, USA). The lysate was mixed with 4 × Laemmli buffer. Total protein extracts were resolved on polyacrylamide gel and then transferred onto nitrocellulose membrane. The following primary antibodies were used: Gapdh (sc-32,233), ID4 (H70) sc-13047, ID4 (B5) sc-365656, HA (12CA5) sc-57592 (Santa Cruz Biotechnology, Dallas, TX, USA); HIF-1A (A300-286A; Bethyl Laboratories); GRN (PA5-29909), EphB2 (PA5-14607), and Mdk (PA5-30601; Thermo Fisher Scientific). Secondary antibody fused with horseradish peroxidase was used for chemiluminescence detection on a UVITEC instrument (Uvitec, Cambridge, UK). VEGFA blocking antibody (AF-293-NA; R&D Systems, Minneapolis, MN, USA) was added to CM and incubated for 30 minutes at room temperature before being used to culture macrophages, following the manufacturer’s instructions.

### RNA isolation, RT-qPCR and TaqMan Low Density Arrays

RNA was isolated with TRIzol reagent (Sigma-Aldrich), and its concentration was measured using a NanoDrop 2000 instrument (NanoDrop Technologies, Wilmington, DE, USA). Reverse transcription was performed with Moloney murine leukemia virus reverse transcriptase (Thermo Fisher Scientific). qPCR was carried out on an ABI PRISM 7500 Fast Sequence Detection System (Applied Biosystems, Foster City, CA, USA). Primers used for PCR analyses are available upon request. The expression values of mRNAs were calculated by the standard curve method and normalised with housekeeping control genes (*GAPDH*, β-actin, H3). qPCR using TaqMan Low Density Arrays (TLDA) Human Angiogenesis (4378725; Thermo Fisher Scientific) was carried out following the manufacturer’s instructions on an ABI PRISM 7900HT Sequence Detection System.

### Angiogenic assay in zebrafish embryo

Four microlitres of CM was mixed with 4 μl of Growth Factor Reduced Matrigel (BD Biosciences) and 0.5 μl of phenol red. The mixture of CM and Matrigel was injected into the perivitelline space of Tg(fli:EGFP) casper zebrafish embryos at 48 hours post-fertilisation. The injection was performed using glass micropipettes with capillaries of 0.75-mm internal diameter. The following parameters were used for the micropipette puller (P-1000; Sutter Instruments, Novato, CA, USA): heat 510, pull 100, velocity 200, time 40, and pressure 500. The parameters of the PicoPump injector (World Precision Instruments, Sarasota, FL, USA) were set to inject 1 nl of CM. Within 24 hours after injection, the neovascular response originating from the developing subintestinal vessels was observed on a fluorescence stereoscope.

### Tube-formation assay

Differentiated U937 cells were transfected with siRNAs directed to GRN mRNA or control siRNAs for 8 hours and subsequently cultured with CM from MDA-MB-468 cells. After 72 hours of culture, CM was collected and used to perform tube-formation assays as described by Pruszko et al. [30].

### Cell viability assay

Viability of U937 cells was assessed using the ATPlite assay (PerkinElmer, Waltham, MA, USA) at the indicated time point and according to the manufacturer’s instructions. Differentiated U937 cells (1 × 10^5^ cells), previously transfected with GRN expression vector, were seeded into 96-well plates and cultured for 48 hours in CM from MDA-MB-468 cells. Luminescence was read by using the EnSpire® Multimode Plate Reader (PerkinElmer).

## Results

### ID4 expression correlates with macrophage recruitment in triple-negative breast cancer

We previously demonstrated that ID4 protein expression is associated with high microvessel density in BC. Mechanistically, ID4 promotes the production of pro-angiogenic cytokines in BC cells, leading to enhanced endothelial cell proliferation and migration [28, 30]. Because the onset of angiogenic switch, identified as the formation of a high-density vessel network, is closely associated with the transition to malignancy and is regulated by infiltrating macrophages in primary mammary tumours [5], we investigated whether ID4 promotes angiogenesis by influencing the behaviour of macrophages. We first evaluated whether any association existed between ID4 protein expression and infiltrating TAMs in human BC by staining a series of 62 TNBCs for ID4 protein and for the widely used macrophage marker CD68 [15, 35]. The choice of TNBC was based on evidence that increased ID4 expression is specific to this subtype, characterised by the absence of oestrogen receptor, PgR and HER2 receptors, and mostly attributable to the BLBC molecular subtype, as reviewed by Baker et al. [23]. Expression levels of ID4 in representative TNBC and BLBC cohorts are shown in Additional file 3: Figure S1. Pathological characteristics of the 62 analysed TNBC cases are included in Additional file 2: Table S1.

**Fig. 1:**
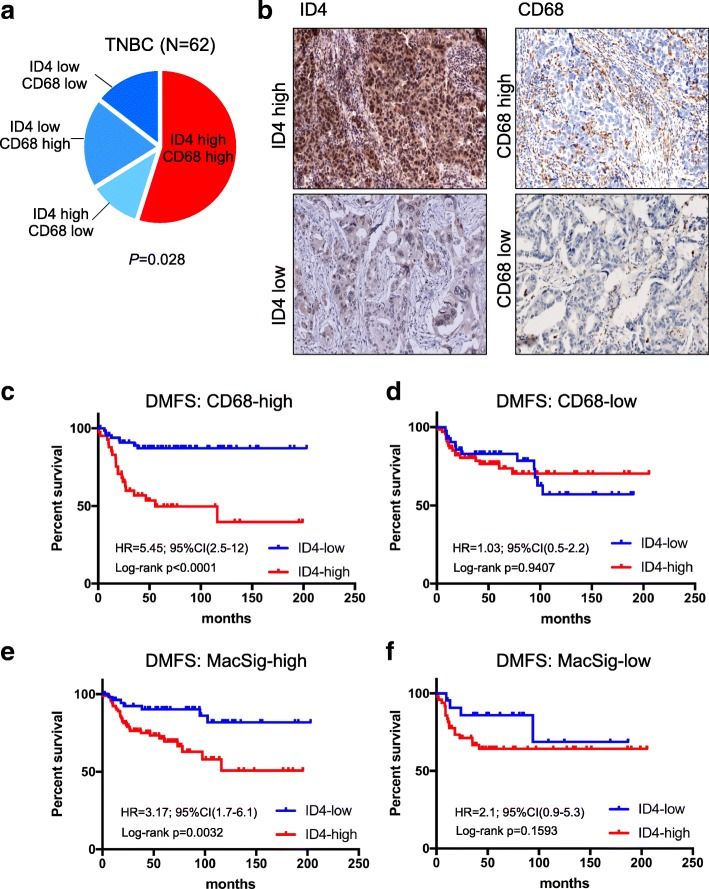
Inhibitor of differentiation 4 (ID4) protein and macrophage marker CD68 are significantly associated in triple-negative breast cancer (TNBC). **a** and **b** A series of 62 TNBC samples was stained for ID4 protein and for the macrophage marker CD68. ID4 protein expression was considered positive when we observed an immunoreaction in the cytoplasm and/or nucleus. Staining intensity was evaluated as follows: 0 negative, 1+ mild, 2+ moderate, 3+ strong. ID4 was considered overexpressed when more than 10% of neoplastic cells presented a strong immunoreaction. CD68 staining was scored as the infiltration density and was evaluated as follows: 0 absent, 1+ mild, 2+ moderate, 3+ dense. **a** Fisher’s exact test demonstrated that high ID4 and CD68 expression are significantly associated (*P* = 0.028). **b** Representative images of TNBC showing high or low protein levels of ID4 or CD68. **c**–**f** Predictive power of *ID4* messenger RNA expression for distant metastasis-free survival (DMFS) (*N* = 232) was evaluated by Kaplan-Meier analysis in basal-like breast cancer (BLBC) showing high or low CD68 (**c** and **d**) or macrophage signature (MacSig) (**e** and **f**) levels. Macrophage signature is composed of eight widely used markers for the mononuclear phagocyte system (CD14, CD105, CD11b, CD68, CD93, CD33, IL4R, and CD163 [37])

In agreement with the literature [18, 28], we observed that ID4 protein was detectable in 75% of the analysed specimens. On the basis of protein expression, we divided the analysed tumours into low expressers (comprising negative tumours and tumours scored as 1+) and high expressers (comprising tumours scored as 2+ and 3+). We observed that high CD68 protein expression was significantly associated with the ID4 high expresser group (*P =* 0.028) (Fig. 1a). Representative images of TNBC showing high or low protein levels of ID4 and CD68 are shown in Fig. 1b. ID4 and CD68 proteins were not associated with other pathological characteristics in this group of patients.

### ID4 expression predicts survival in tumours highly infiltrated by macrophages

High levels of ID4 expression have been correlated to decreased survival in TNBC and BLBC [17, 20, 21]. Macrophage infiltration has been correlated to angiogenesis in BC, but the study of its prognostic significance has led to contradictory results, probably because of the existence of various intratumoural macrophage populations with different properties [12].

To evaluate ID4 prognostic power in relation to macrophage infiltration, we interrogated the Kaplan-Meier Plotter database (www.kmplot.com) [36], which contains a compendium of studies with gene expression and relative survival data for BLBCs. Interestingly, we observed that high ID4 expression was strongly associated with low probability of DMFS (*n* = 232) and OS (*n* = 241), specifically in the group of tumours characterised by high expression of CD68 (and therefore highly infiltrated by macrophages) (Fig. 1c and Additional file 4: Table S2), whereas no association of ID4 with survival was present in the low-CD68 group (Fig. 1d and Additional file 4: Table S2). A similar result was obtained when a macrophage signature comprising a subset of eight widely used markers (CD14, CD105, CD11b, CD68, CD93, CD33, IL-4R and CD163) for the mononuclear phagocyte system [37] was used to identify tumours highly infiltrated by macrophages (Fig. 1e and f and Additional file 5: Table S3). Analysis of gene expression data from The Cancer Genome Atlas (TCGA) cohort of BLBC confirmed that high ID4 expression is associated to low probability of overall survival specifically in the CD68-high and macrophage signature (MacSig)-high groups (Additional file 6: Figure S2a–d). The TCGA cohort allowed us also to assess that ID4 and CD68 do not associate with the clinical variables T, N and G (as observed in the TNBC cohort analysed by IHC and described in the previous paragraph), whereas ID4 significantly associates with mutated *TP53* status (Additional file 6: Figure S2e). Moreover, because none of the considered patients from the TCGA cohort received neoadjuvant treatment, we can assert that the observed associations are independent of particular treatment regimens. These results indicated that the combination of ID4 and macrophage markers represents a powerful predictive indicator in BLBC.

### ID4 expression in breast cancer cells enhances macrophage motility

**Fig. 2:**
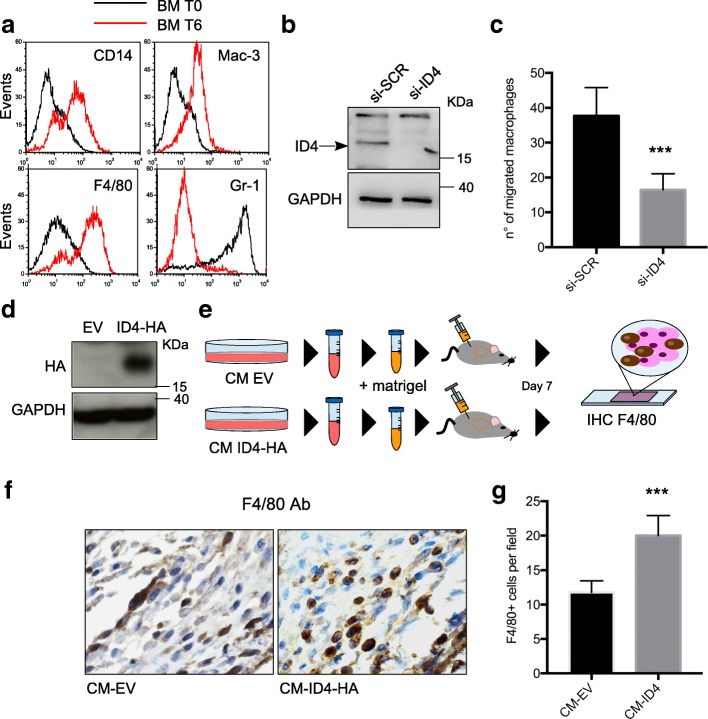
Inhibitor of differentiation 4 (ID4) expression in breast cancer cells enhances macrophage motility. **a** Control of differentiation markers by fluorescence-activated cell sorting analysis in mouse bone marrow-derived macrophages before (T0) and after (T6) culturing in CSF1-rich medium (L929) for 6 days. **b** Efficiency of ID4 depletion in the SKBR3 cells used for migration assays, evaluated by Western blotting. **c** Migratory capacity of mouse bone marrow-derived macrophages in response to SKBR3 breast cancer cells, depleted (si-ID4) or not depleted (si-SCR) of ID4 expression, evaluated by Transwell assay. **d** Efficiency of hemagglutinin (HA)-tagged ID4 overexpression (ID4-HA) compared with that of empty vector transfection (EV) evaluated by using an anti-HA antibody in Western blot analysis. ID4-HA and EV MDA-MB-468 cells were used to prepare conditioned media (CM) for in vivo Matrigel assay. **e** Schematic representation of Matrigel assay. **f** and **g** IHC analysis of mouse macrophage marker F4/80 on Matrigel plugs containing the indicated CM and recovered from mouse flanks at day 7 after inoculation. Counts of F4/80^+^ cells are indicated in (**g**). Results from at least three biological replicates are shown. Data are presented as mean ± SEM. ***P < 0.0005 calculated by two-tailed *t* test

On the basis of the observed association between ID4 protein expression and TAMs, we wondered whether ID4 expression in BC cells influences macrophage recruitment. To address this, CD34^+^ progenitors from mouse bone marrow were isolated, differentiated in vitro to macrophages (Fig. 2a), and evaluated for their migratory capacity in response to BC cells with ID4 expression depleted or not (Fig. 2b–c). As shown in Fig. 2c, a lower number of macrophages migrated towards ID4-depleted (si-ID4) BC cells than that for control (si-SCR) cells.

To evaluate if ID4 expression in BC cells influences the recruitment of macrophages in vivo, we performed Matrigel assays. Briefly, Matrigel plugs containing CM from MDA-MB-468 BC cells, transfected with an expression vector for HA-tagged ID4 or an empty vector (Fig. 2d and e), were inoculated subcutaneously in mouse flanks and recovered after 7 days. According to previous reports [38, 39], IHC staining of Matrigel plugs with mouse monocyte/macrophage marker F4/80 showed the presence of F4/80^+^ cells within regions of massive cellular infiltration inside the Matrigel. A higher number of F4/80^+^ cells was observed in plugs containing CM from ID4-overexpressing cells than that in control plugs (Fig. 2f–g).

### ID4 expression in breast cancer cells modulates the activation of a pro-angiogenic programme in macrophages

**Fig. 3:**
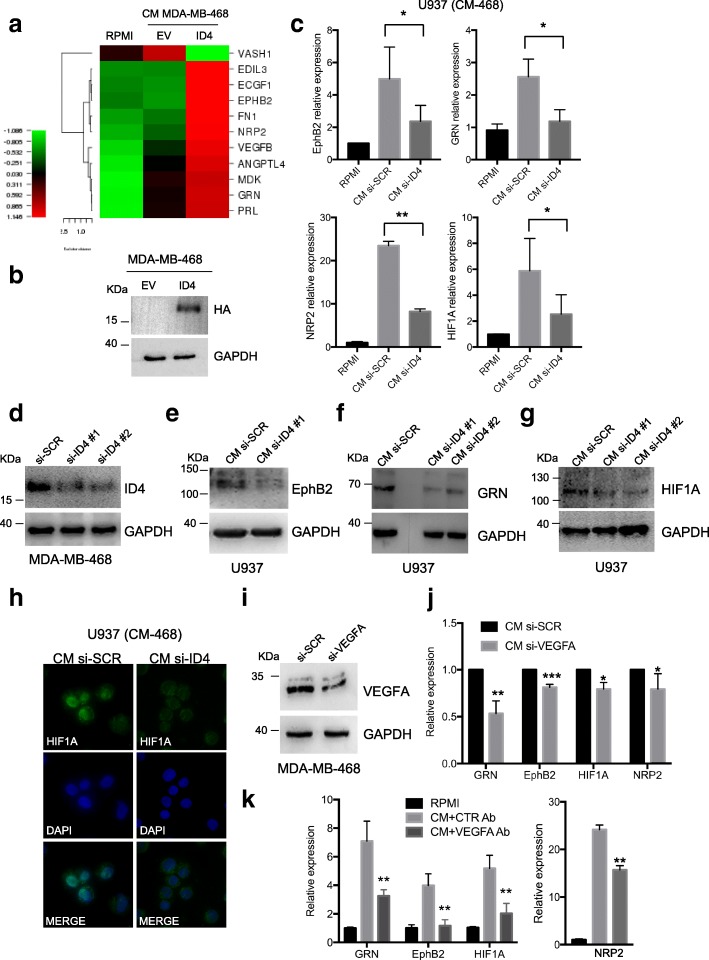
Inhibitor of differentiation 4 (ID4) expression in breast cancer cells leads to the activation of an angiogenic programme in macrophages. **a** Expression matrix representing a panel of angiogenic factors evaluated using TaqMan Low-Density Arrays (TLDA) in macrophages obtained by 1,25-dihydroxyvitamin D_3_ (VitD3)-mediated differentiation of HL60 cells and subsequently cultured in RPMI medium or in conditioned media (CM) from control (EV) or ID4-overexpressing (ID4) MDA-MB-468 breast cancer cells. **b** Western blot showing ID4-HA overexpression in MDA-MB-468 cells. **c** Selected genes modulated in the arrays were evaluated by RT-qPCR in macrophages obtained from VitD3-mediated differentiation of U937 cells and subsequently cultivated in RPMI medium (CTR) or in CM from control (CM si-SCR) or ID4-depleted (CM si-ID4) MDA-MB-468 cells. **d** Western blot analysis showing the level of ID4 protein after transfection of the indicated small interfering RNAs (siRNAs) in MDA-MB-468 cells. **e**–**g** Western blot analysis of ephrin B2 (EphB2), granulin (GRN) and hypoxia-inducible factor (HIF)-1A proteins in differentiated U937 cells cultured in CM si-SCR or CM si-ID4 from MDA-MB-468 cells. **h** Immunofluorescence analysis of HIF-1A protein performed in differentiated U937 cells cultured in the presence of CM si-SCR or CM si-ID4 from MDA-MB-468 cells. **i** Western blotting showing the efficiency of vascular endothelial growth factor A (VEGFA) depletion by siRNA transfection in MDA-MB-468 cells used to prepare CM used in experiments shown in (**j**). **j** RT-qPCR analysis of the indicated messenger RNAs in U937 macrophages cultivated in the presence of CM from control (si-SCR) or VEGFA-depleted (si-VEGFA) MDA-MB-468 cells. **k** RT-qPCR analysis of the indicated genes in differentiated U937 cells cultivated in RPMI medium or in CM from MDA-MB-468 cells in the presence of VEGFA blocking antibody (Ab) or a control Ab. Specifically, VEGFA blocking Ab or control Ab were incubated with CM for 30 minutes at room temperature and CM plus Ab was subsequently used to culture U937 cells for 48 hours. Results from at least three biological replicates are shown. Data are presented as mean ± SEM. *P < 0.05, **P < 0.005, ***P < 0.0005 calculated by two-tailed *t* test

Because one of the major activities exerted by TAMs is the promotion of angiogenesis, we next analysed whether ID4 expression in BC cells affects the expression of angiogenic genes in macrophages. To this end, we took advantage of a TLDA containing probes for a panel of 94 angiogenesis-related genes. Macrophages obtained from differentiation of HL60 cells [40, 41], cultured with CM from MDA-MB-468 cells transfected with an ID4 expression vector (ID4) or an empty vector (EV), were evaluated along with control macrophages cultured in RPMI medium (Fig. 3a and b). In this experimental setting, we detected 36 expressed genes, 11 of which were modulated in an ID4-dependent manner (1 downregulated and 10 upregulated genes) (Additional file 4: Table S3). The ID4-dependent paracrine induction in macrophages of a subset of these genes, comprising ephrin B2 (*EPHB2*), midkine (*MDK*), *EDIL3* and *GRN*, was validated by RT-qPCR (Additional file 7: Figure S4a) and Western blotting (Additional file 7: Figure S4b). We verified that ID4 overexpression did not affect the expression of these genes in MDA-MB-468 cells (Additional file 7: Figure S4a, right panel).

Moreover, using an additional macrophage cell line (U937), we observed that the expression of selected ID4-dependent angiogenesis-related genes (*EPHB2*, *GRN* and *NRP2*) was induced in macrophages cultivated in CM compared with RPMI medium (Fig. 3c); as expected, this induction was impaired when CM was derived from si-ID4 BC cells (Fig. 3c–f). Interestingly, analysis of HIF-1A, a master regulator of angiogenesis, revealed that the expression of this transcription factor in macrophages depends on the level of ID4 expression in BC cells (Fig. 3c, g and h and Additional file 7: Figure S4c). Altogether, these results showed that high ID4 expression in BC cells is associated with the activation of a pro-angiogenic programme in macrophages.

Because the expression of the angiogenesis-related genes in macrophages depends on the expression of ID4 in BC cells, we reasoned that a soluble factor, secreted in an ID4-dependent manner from BC cells, is probably responsible for the observed gene expression reprogramming of macrophages. In this regard, we recently reported that ID4 protein promotes the synthesis of pro-angiogenic isoforms of VEGFA at the expense of the anti-angiogenic ones in BC cells [30]. We then explored whether VEGFA was responsible for the observed effects. We first cultured differentiated U937 cells in CM from VEGFA-depleted (si-VEGFA) or control (si-SCR) BC cells. Analysis of a panel of angiogenesis-related factors evidenced a partial decrease of their expression after VEGFA depletion (Fig. 3I and j). Next, we observed that the addition of VEGFA blocking antibody to the CM from BC cells subsequently used to culture U937 cells partially impaired the induction of this panel of angiogenesis-related factors (Fig. 3k). These results indicate that ID4-dependent gene expression modulation in macrophages is at least in part under the control of VEGFA signalling.

### ID4 expression in breast cancer cells downregulates anti-angiogenic microRNAs in macrophages

It has been extensively reported that the angiogenic programme is tightly controlled also at the post-transcriptional level by miRNAs in cancer. To explore whether the ID4-dependent reprogramming of macrophages also involved miRNAs, we evaluated the expression of members of the miR-15/107 group, which were previously correlated to angiogenesis in vertebrates and reported to target GRN and HIF-1B [42–47].

**Fig. 4:**
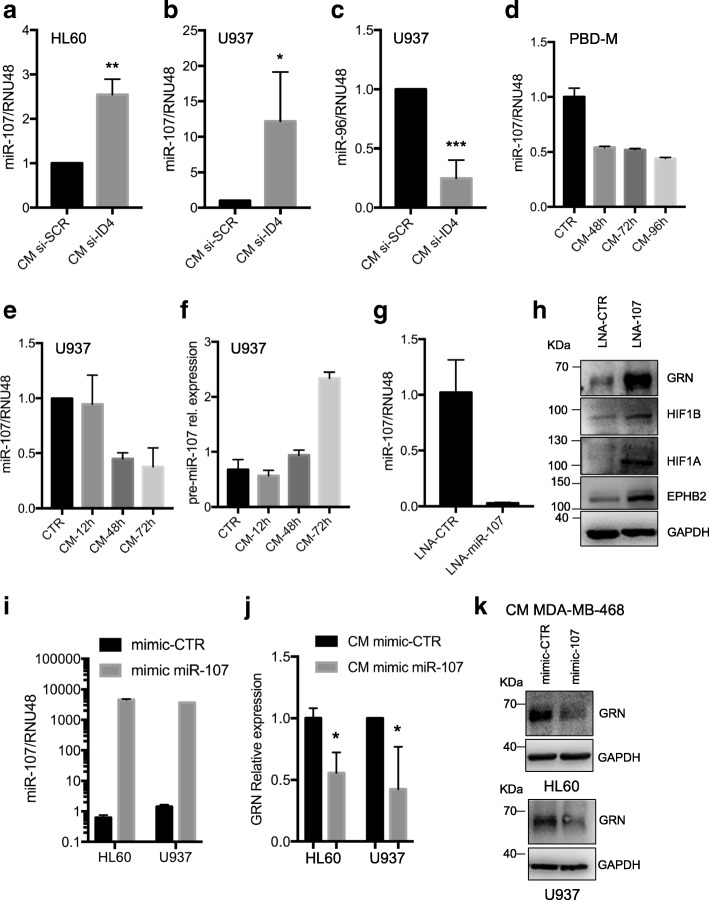
Inhibitor of differentiation 4 (ID4) expression in breast cancer cells leads to the paracrine downregulation of miR-107 in macrophages. **a** and **b** RT-qPCR analysis to evaluate the expression of miR-107 in macrophages obtained from 1,25-dihydroxyvitamin D_3_ (VitD3)-mediated differentiation of HL60 (**a**) and U937 (**b**) cells and subsequently cultivated in conditioned media (CM) from control (si-SCR) or ID4-depleted (si-ID4) MDA-MB-468 cells. **c** RT-qPCR for miR-96 in U937-derived macrophages as in (**b**). **d** and **e** RT-qPCR analysis of miR-107 in peripheral blood-derived macrophages (PBD-M) (**d**) and U937-derived (**e**) macrophages cultivated in RPMI medium (CTR) or in CM from, respectively, SKBR3 and MDA-MB-468 cells for the indicated time points. **f** RT-qPCR for pre-miR-107 in U937 cells as in (**e**). **g** RT-qPCR analysis of miR-107 levels in differentiated U937 cells transfected with locked nucleic acid (LNA) antisense oligonucleotide directed to miR-107. **h** Western blot analysis of the indicated proteins in differentiated U937 cells transfected with LNA antisense oligonucleotide directed to miR-107. **i** and **j** miR-107 (**i**) and granulin (GRN) (**j**) expression levels evaluated by RT-qPCR in HL60 and U937 cells transfected with control mimic or miR-107 mimic oligonucleotides. **k** Western blot analysis of GRN in HL60 and U937 cells transfected with control mimic or miR-107 mimic oligonucleotides. Results from at least three biological replicates are shown. Data are presented as mean ± SEM. *P < 0.05, **P < 0.005, ***P < 0.0005 calculated by two-tailed *t* test.

We observed that miR-107, miR-15b and miR-195 are downregulated in macrophages cultured with CM from ID4-overexpressing BC cells (CM ID4) compared with macrophages cultured with CM from BC cells with control empty vector (CM EV) (Additional file 5: Figure S5a). On the contrary, expression of these miRNAs was recovered in the presence of CM from si-ID4 BC cells in two macrophage cell lines (Fig. 4a and b and Additional file 8: Figure S5b–e). We evaluated the expression of miR-96, which exhibits oncogenic activity in BC [48], as a control, and we observed that it shows a trend opposite to that of miR-107 (Fig. 4c). Recovery of miR-107, miR-15b and miR-195 expression was also observed in U937 cells cultured in the presence of CM from VEGFA-depleted BC cells (Additional file 8: Figure S5f), indicating that VEGFA signalling also controls, at least in part, miRNA expression in TAMs.

Time-course analysis of macrophages cultured with CM from BC cells revealed downregulation of these miRNAs (Fig. 4d and e and Additional file 8: Figure S5f). Analysis of pre-miR-107 expression in the same conditions highlighted that decrease of mature miR-107 was accompanied by an accumulation of its precursor (Fig. 4f), suggesting an inhibition of the processing of this miRNA in the presence of CM from BC cells. Altogether, these results indicated that expression of ID4 in BC cells leads to a paracrine downregulation of miR-107, miR-15b and miR-195 in macrophages.

Next, we focused on miR-107, which shows the strongest ID4-dependent paracrine downregulation in macrophages, and evaluated whether it affects the expression of GRN and HIF-1B, two well-established targets [44, 49]. To this end, we inhibited miR-107 in U937 cells by transfecting an LNA oligonucleotide (Fig. 4g). As shown in Fig. 4h, miR-107 inhibition recovered GRN and HIF-1B protein expression, mimicking the effect of si-ID4 BC-derived CM. We also observed induced protein expression of EphB2 and HIF-1A (Fig. 4h), which, as the majority of the angiogenesis-related factors that are activated in an ID4-dependent paracrine manner in macrophages, are predicted to be targeted by the miR-15/107 group members (Additional file 5: Table S3).

To further investigate the relevance of miR-107 downregulation associated with CM, we overexpressed miR-107 using mimic oligonucleotides in macrophages cultured with CM from MDA-MB-468 BC cells (Fig. 4i). As shown in Fig. 4j and k, the forced expression of miR-107 led to decreased GRN mRNA and protein levels. Similar results were observed for HIF-1A (Additional file 8: Figure S5g and h). Our results indicated that the expression of angiogenesis-related genes is strictly controlled by the activity of the ID4-dependent miR-107 in macrophages.

### Granulin expression markedly increases the angiogenic potential of macrophages

Among the ID4-dependent angiogenesis-related genes upregulated in macrophages, *GRN* particularly attracted our attention, because this growth factor is specifically expressed in TNBC and BLBC [50] and has recently been correlated to tumour angiogenesis in mesothelioma [51]. In macrophages, GRN has been reported to control cytokine production [32], but its effect on the angiogenic potential of these cells has not been explored yet.

**Fig. 5:**
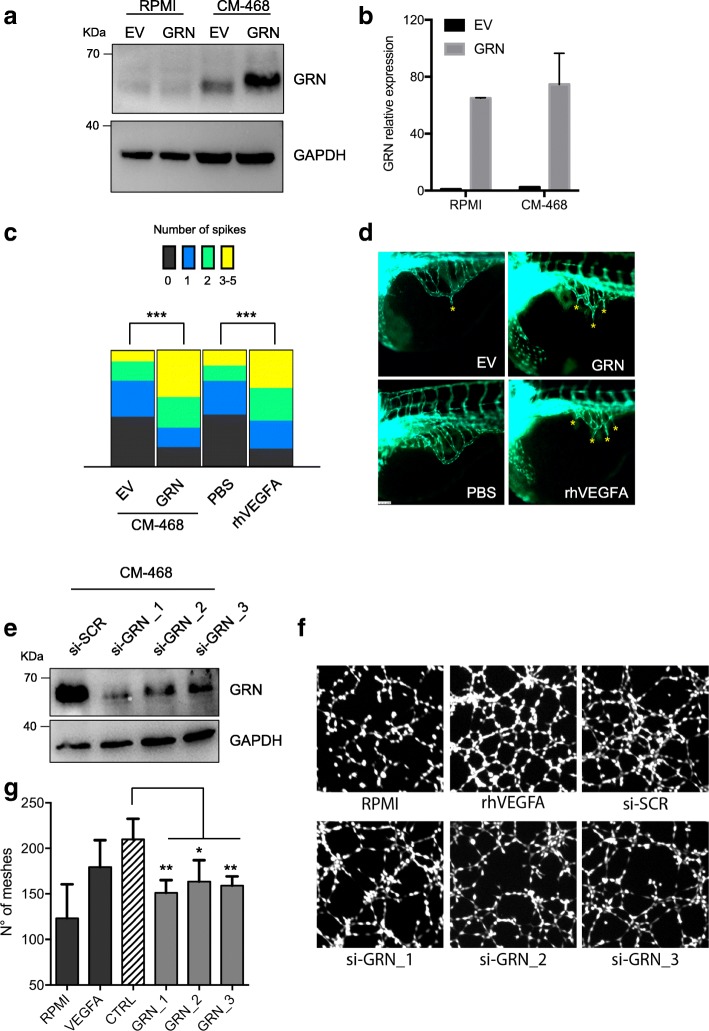
Modulation of granulin (GRN) expression affects the angiogenic potential of macrophages. **a** Western blot analysis of GRN in U937 cells transfected with a GRN expression vector or an empty vector (EV) and cultured in the presence of RPMI medium or conditioned media (CM) from MDA-MB-468 cells. **b** RT-qPCR analysis of *GRN* messenger RNA levels in the same experimental conditions described in (**a**). **c** CM from the indicated experimental conditions were injected into the perivitelline space of zebrafish embryos, and neovascular response originating from the developing subintestinal plexus was evaluated. Injection of PBS alone or PBS supplemented with recombinant vascular endothelial growth factor A (rhVEGFA) was used, respectively, as negative and positive controls. Spikes sprouting from subintestinal plexus were counted in at least 42 embryos per condition. Graph shows the distribution of the population of embryos evaluated for each condition. Representative images are shown in (**d**). Significance was evaluated by one-way analysis of variance followed by Sidak’s multiple comparisons test using GraphPad software (GraphPad, La Jolla, CA, USA). ***P < 0.0005. **e** Western blot analysis of GRN in U937 cells transfected for 8 hours with control small interfering RNA (si-SCR) or three different GRN-targeting siRNAs (si-GRN_#1,_#2,_#3) and subsequently cultured in the presence of CM from MDA-MB-468 cells for 72 hours. **f** and **g** Tube-formation assays involving EA.Hy926 endothelial cells performed in the presence of CM from the conditions indicated in (**e**). RPMI medium, supplemented (rhVEGFA) or not (RPMI) with recombinant VEGFA, was used as a positive or negative control, respectively. Data are presented as mean ± SEM. *P < 0.05, **P < 0.005 calculated by two-tailed *t* test

To evaluate the ability of GRN to confer angiogenic potential to macrophages, we performed in vivo angiogenic assays. To this end, a full-length GRN expression vector, containing 5′- and 3′-UTRs, or control EV was transfected in U937-derived macrophages, which were then cultured with RPMI or CM from MDA-MB-468 cells. As shown in Fig. 5a and b, although *GRN* mRNA expression levels were comparable between RPMI and CM conditions, GRN protein overexpression was observed only in macrophages cultured with CM. This result further underlined that GRN expression in macrophages is strictly controlled at the translational level and that its protein expression is obtained only in the presence of CM, possibly as a consequence of miR-107 downregulation (as shown in Fig. 4d and e).

We then evaluated the angiogenic potential of macrophages transfected with GRN or EV and cultured in CM from MDA-MB-468 cells using transgenic zebrafish embryos expressing enhanced green fluorescent protein in the entire vasculature. Specifically, Matrigel plugs containing CM from each experimental condition were injected into the perivitelline space of zebrafish embryos, and neovascular response originating from the developing subintestinal plexus was evaluated. Injection of Matrigel plugs containing PBS alone or PBS supplemented with recombinant VEGFA (rhVEGFA) was used as a negative and positive control, respectively. As shown in Fig. 5c and d, we observed a greater number of embryos presenting two or more spikes sprouting from subintestinal plexus in the GRN overexpression condition compared with that in the EV condition. Accordingly, in the GRN overexpression condition, we also observed a reduced number of embryos showing one or no spikes (Fig. 5c and d). No effects on macrophage viability and differentiation were observed in the presence of GRN overexpression (Additional file 9: Figure S6).

Next, we evaluated the effect of GRN depletion on macrophage angiogenic potential. To this end, we transfected siRNAs directed to GRN or control siRNAs (si-SCR) in U937-derived macrophages, which were then cultured with CM from MDA-MB-468 cells (Fig. 5e). CM from each experimental condition was then evaluated in tube-formation assays involving the growth of endothelial cells. Conditions with RPMI medium supplemented or not with rhVEGFA were included as positive and negative controls, respectively. As shown in Fig. 5f and g, GRN depletion led to the significant decrease of tube-formation potential.

## Discussion

**Fig. 6:**
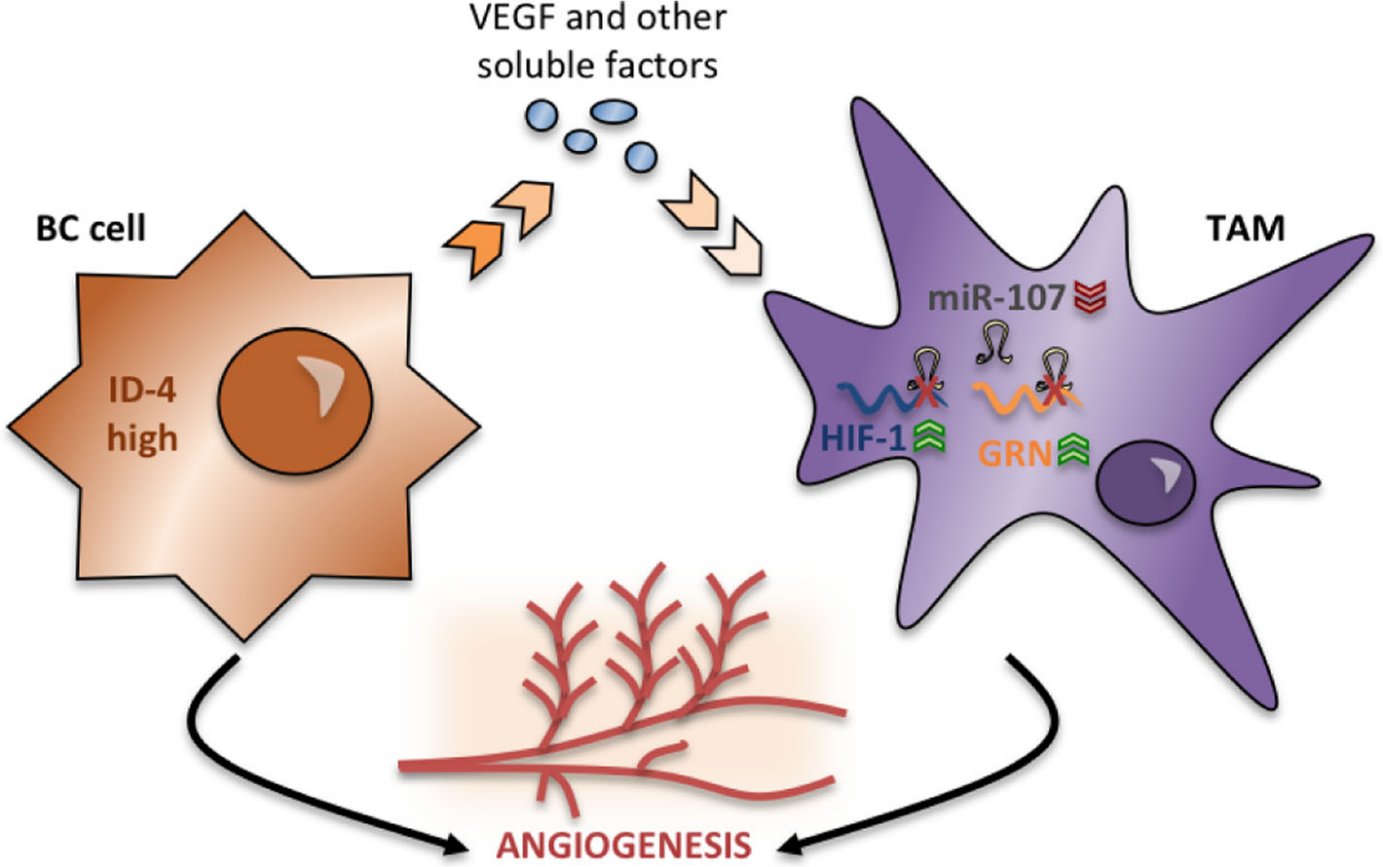
Summary scheme of the identified paracrine signalling from breast cancer (BC) cells to macrophages. Briefly, breast cancer cells expressing high levels of inhibitor of differentiation 4 (ID4) protein produce vascular endothelial growth factor A (VEGFA) and other factors implicated in the induction of an angiogenic programme in neighbouring macrophages. In parallel to the induction of angiogenesis-related messenger RNAs, we observed a decrease of miR-15b/107 group members, with consequent release of the expression of its targets, as transcription factor hypoxia-inducible factor (HIF)-1A and granulin (GRN). *TAM* Tumour-associated macrophage

In this study, we demonstrated that expression of ID4 in BC cells is an important determinant of TAM behaviour. High ID4 expression in BC cells indeed is able to cause not only macrophage recruitment but also the reprogramming of macrophage gene expression (Fig. 6). Specifically, we observed that ID4 modulates a panel of angiogenesis-associated factors, among which there is an important regulator of inflammation, GRN [32, 52].

It has been reported that GRN directly binds to tumour necrosis factor (TNF) receptors and counteracts the TNF-mediated inflammatory signalling pathway. GRN also induces regulatory T-cell populations and IL-10 production and inhibits CXCL9 and CXCL10 chemokine release. It will be interesting to evaluate in further studies whether ID4-dependent GRN induction occurring in macrophages has an immunomodulatory effect in BC. Analysis of tumour tissues from a cohort of patients with BC revealed that high GRN expression correlated with the most aggressive triple-negative BLBC and reduced patient survival [50].

In addition to the angiogenesis-related factors induced in an ID4-dependent manner, we observed in macrophages a similar increase of transcription factor HIF-1. HIF-1 has previously been reported to be strongly involved in TAM pro-tumourigenic activities. Of note, the majority of the angiogenesis-related factors that we have identified present HIF-1 consensus sequences in their promoter regions and therefore could be subjected to HIF-1-dependent transactivation (Additional file 9: Table S3).

Another important aspect of this study is the identification of VEGF, whose isoform synthesis is controlled by ID4 in BC cells [30], as one of the soluble factors participating in the paracrine activation of the angiogenic programme in co-cultured macrophages. We have indeed recently identified that VEGFA isoform expression is controlled in BC cells by a ribonucleoprotein complex containing, in addition to ID4, the splicing factor SRSF1, the mutant p53 protein and the long non-coding RNA *MALAT1* [30]. This complex favours the production of VEGF121 and VEGF165 isoforms. Because the addition of blocking antibodies directed to VEGFA in CM from BC cells significantly reduced the angiogenic programme activation in macrophages, it is highly probable that this programme depends on the ribonucleoprotein complex controlling VEGFA expression in BC cells. Interestingly, we showed that blocking of VEGFA prevents CM-dependent activation of *EPHB2* and *NRP2*, among others. Of note, both these genes have been reported to participate in the enhancement of VEGFA signalling through VEGFR2 [53–55]. Activation of *EPHB2* and *NRP2* then could represent a mechanism for VEGFA signalling amplification in macrophages, because an increase of these molecules will probably lead to a more efficient response to the VEGFA present in the CM (in our experimental system) and in the in vivo tumour microenvironment.

Finally, we identified an additional layer of control of the angiogenesis-related genes in macrophages (i.e., the post-transcriptional layer). Indeed, among the identified angiogenesis-related factors, HIF-1 and GRN are interestingly controlled by miR-107, whose expression is downregulated in macrophages in an ID4-dependent manner. miR-107 and another miRNA of this family (miR-195) that we have found downregulated in an ID4-dependent manner in macrophages were previously shown to have tumour-suppressive properties in BC [56–59]. Our study elucidates a novel role for these miRNAs in the control of the angiogenic programme in TAMs.

## Conclusion

Taken together, our results reveal that ID4 protein, previously shown to control the stem-like phenotype of normal and transformed mammary epithelial cells, also controls the angiogenic potential in breast cancer through the modulation of tumor-associated macrophage activity. The identified paracrine signaling may represent a promising basis for the development of therapies aimed at disrupting the cross-talk between cancer cells and tumor stroma.

## Additional files

Additional file 1: **Figure S3** Growth curve of MDA-MB-468 cells depleted (si-ID4) or not (si-SCR) of ID4 expression by siRNA transfection (**a**). Cells were transfected for 16 hours, and then equal numbers of cells were plated and counted at the indicated time points. Efficiency of ID4 depletion at 48 hours and 72 hours was evaluated by Western blotting (**b**). (PDF 4554 kb)

Additional file 2: **Table S1** Characteristics of patients selected for the analysis of ID4 protein expression. (DOCX 17 kb)

Additional file 3: **Figure S1.** Comparison of ID4 mRNA expression in basal-like breast cancer (BLBC) and triple-negative breast cancer (TNBC) versus all other breast cancer subtypes (Others) in the indicated representative datasets [19, 22, 60]. (PDF 143 kb)

Additional file 4: **Table S2** Predictive power of ID4, CD68 and the macrophage signature (MacSig) comprising eight widely used markers (CD14, CD105, CD11b, CD68, CD93, CD33, IL4R, CD163) for the mononuclear phagocyte system [37]. Analysis was performed using datasets deposited in the KMplot database [36]. *DMFS* Distant metastasis-free survival, *OS* Overall survival. (DOCX 21 kb)

Additional file 5: **Table S3** mRNAs modulated in an ID4-dependent manner in differentiated HL60 cells cultured with conditioned medium from control (CM EV) or ID4-overexpressing (CM ID4) MDA-MB-468 cells. The presence of HIF-1 consensus sequences on promoters was evaluated using the LASAGNA-Search web tool (http://biogrid-lasagna.engr.uconn.edu/lasagna_search/). The presence of putative binding sites for miR-107, miR-15b and miR-195 on 3′-UTR or coding (CDS) sequences of mRNAs was evaluated using the miRWalk analysis tool (http://zmf.umm.uni-heidelberg.de/apps/zmf/mirwalk2/) by selecting the following databases: (1) 3′-UTR analysis = miRWalk, miRanda, miRDB, miRNAMap, Pictar2, RNA22, RNAhybrid, TargetScan; and (2) CDS analysis = miRWalk, miRanda, RNA22, RNAhybrid, TargetScan. (DOCX 22 kb)

Additional file 6: **Figure S2.** Predictive power of *ID4* mRNA expression for overall survival (OS) was evaluated by Kaplan-Meier analysis on the TCGA cohort in BLBCs showing high or low CD68 (**a** and **b**) or macrophage signature (MacSig) (**c** and **d**) levels. Macrophage signature is composed of eight widely used markers for the mononuclear phagocyte system (CD14, CD105, CD11b, CD68, CD93, CD33, IL4R and CD163 [37]). **e** Evaluation of association between ID4 or CD68 and the pathological variables T, N, G and *TP53* status in the BLBCs from the TCGA cohort. (PDF 4464 kb)

Additional file 7: **Figure S4 a** Modulation of selected genes modulated in the TLDA was validated by RT-qPCR in differentiated HL60 cells cultured in CM from ID4-overexpressing (CM ID4-HA) or control (CM EV) MDA-MB-468 cells (left panel). The same transcripts were analysed in MDA-MB-468 cells transfected with ID4-HA expression vector (ID4-HA) or control empty vector (EV) (right panel). **b** Expression of EphB2, MDK and GRN protein evaluated by Western blotting on lysates from differentiated HL60 cells cultured as in (**a**); secreted GRN (sGRN) was evaluated on CM from differentiated HL60 cells in the same conditions. **c** HIF1A protein expression evaluated by Western blotting in differentiated U937 cells cultured in RPMI medium or in CM from SKBR3 cells stably interfered for ID4 expression (sh-ID4) or control cells (sh-CTR). (PDF 1320 kb)

Additional file 8: **Figure S5 a** Expression of miR-107, miR-15b and miR-195 in differentiated HL60 cells cultured with CM from control (CM EV) or ID4-overexpressing (CM ID4) MDA-MB-468 cells. **b**–**e** Expression of miR-15b and miR-195 in HL60 and U937 cells cultured with CM from control (si-SCR) or ID4-depleted (si-ID4) BC cells. **f** miR-107, miR-15b and miR-195 expression evaluated by RT-qPCR in differentiated U937 cells cultured with CM from MDA-MB-468 cells depleted or not of VEGFA expression. VEGFA interference efficiency is shown in Fig. 3i. **g** Expression levels of miR-15b and miR-195 in differentiated U937 cells cultivated in RPMI medium (CTR) or CM from MDA-MB-468 cells for the indicated time points. **h** and **i** HIF1A mRNA (**h**) and protein (**i**) expression evaluated, respectively, by RT-qPCR and immunofluorescence in differentiated U937 cells transfected with control mimic or miR-107 mimic and cultured in the presence of CM from MDA-MB-468 cells for 48 hours. (PDF 2150 kb)

Additional file 9: **Figure S6** Differentiated U937 cells transfected with an empty vector (EV) or a granulin (GRN) expression vector and subsequently cultivated in the presence of CM from MDA-MB-468 cells were evaluated for their differentiation state (percentage of CD11b^+^ cells) (**a**) and for their viability (**b**) by, respectively, FACS analysis and ATPlite assay at the indicated time points after CM addition. **c** Overexpression of GRN evaluated by Western blotting. (PDF 141 kb)

## Abbreviations

APC: Allophycocyanin
BC: Breast cancer
BLBC: Basal-like breast cancer
CM: Conditioned media
DAB: Diaminobenzidine
DMFS: Distant metastasis-free survival
EV: Empty vector
FACS: Fluorescence-activated cell sorting
GRN: Granulin
HA: Hemagglutinin
HIF: Hypoxia-inducible factor
ID: Inhibitors of differentiation
LNA: Locked nucleic acid
mAb: Monoclonal antibodies
MacSig: Macrophage signature
miRNA, miR: MicroRNA
mRNA: Messenger RNA
OS: Overall survival
RT: Room temperature
si-ID4: ID4-depleted breast cancer cells
siRNA: Small interfering RNA
si-SCR: Control breast cancer cells
TAM: Tumour-associated macrophage
TLDA: TaqMan Low Density Array
TNBC: Triple-negative breast cancer
TNF: Tumour necrosis factor
UTR: Untranslated region
VitD3: 1,25-dihydroxyvitamin D_3_
