# Supplementary figures and images for: Expression of ID4 protein in breast cancer cells induces reprogramming of tumour-associated macrophages
Source: Breast Cancer Res. 2018 Jun 19;20:59. doi: 10.1186/s13058-018-0990-2 (PMC6009061; doi:10.1186/s13058-018-0990-2)

Figure S3

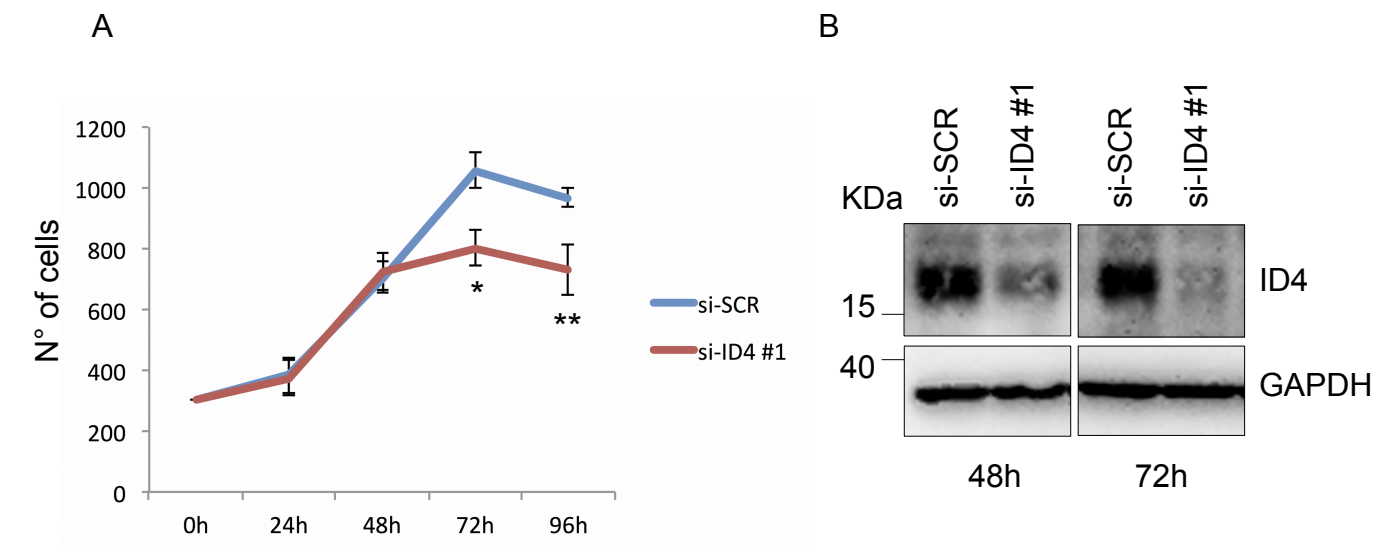

Supplement: Supplementary file 1 — Figure S3 Growth curve of MDA-MB-468 cells depleted (si-ID4) or not (si-SCR) of ID4 expression by siRNA transfection (a). Cells were transfected for 16 hours, and then equal numbers of cells were plated and counted at the indicated time points. Efficiency of ID4 depletion at 48 hours and 72 hours was evaluated by Western blotting (b). (PDF 4554 kb) [file 13058_2018_990_MOESM1_ESM.pdf]

Figure S1

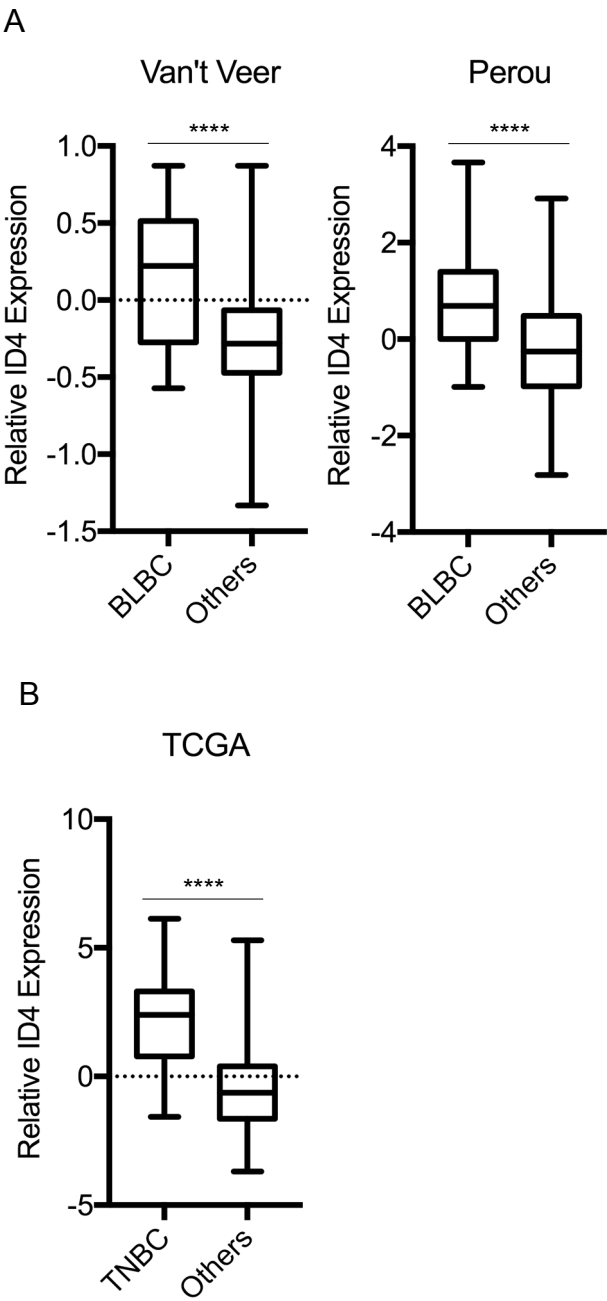

Supplement: Supplementary file 3 — Figure S1. Comparison of ID4 mRNA expression in basal-like breast cancer (BLBC) and triple-negative breast cancer (TNBC) versus all other breast cancer subtypes (Others) in the indicated representative datasets [19, 22, 60]. (PDF 143 kb) [file 13058_2018_990_MOESM3_ESM.pdf]

Figure S2

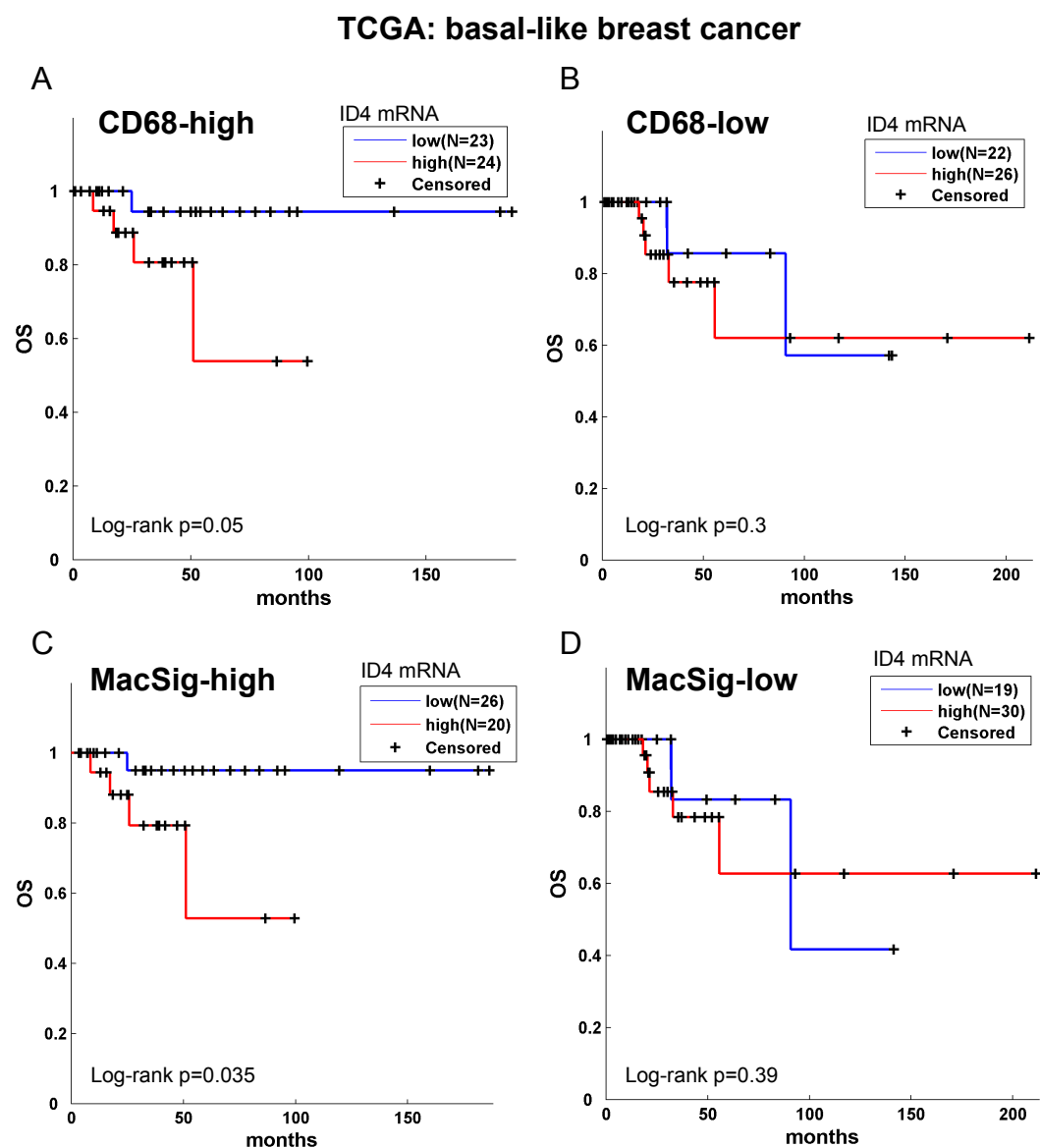

E

|                | OR[CI95%]\pvalue     |                      |                      |                      |
|----------------|----------------------|----------------------|----------------------|----------------------|
| Gene symbol ID | T                    | N                    | G                    | mutP53               |
| ID4 3400       | 1.06[0.76-1.4]\0.72  | 1.05[0.86-1.27]\0.60 | 0.89[0.72-1.09]\0.26 | 1.38[1.06-1.81]\0.01 |
| CD68 968       | 1.02[0.52-2.00]\0.93 | 0.74[0.48-1.12]\0.16 | 1.08[0.71-1.64]\0.69 | 0.79[0.47-1.35]\0.40 |

Supplement: Supplementary file 6 — Figure S2. Predictive power of ID4 mRNA expression for overall survival (OS) was evaluated by Kaplan-Meier analysis on the TCGA cohort in BLBCs showing high or low CD68 (a and b) or macrophage signature (MacSig) (c and d) levels. Macrophage signature is composed of eight widely used markers for the mononuclear phagocyte system (CD14, CD105, CD11b, CD68, CD93, CD33, IL4R and CD163 [37]). e Evaluation of association between ID4 or CD68 and the pathological variables T, N, G and TP53 status in the BLBCs from the TCGA cohort. (PDF 4464 kb) [file 13058_2018_990_MOESM6_ESM.pdf]

Figure S4

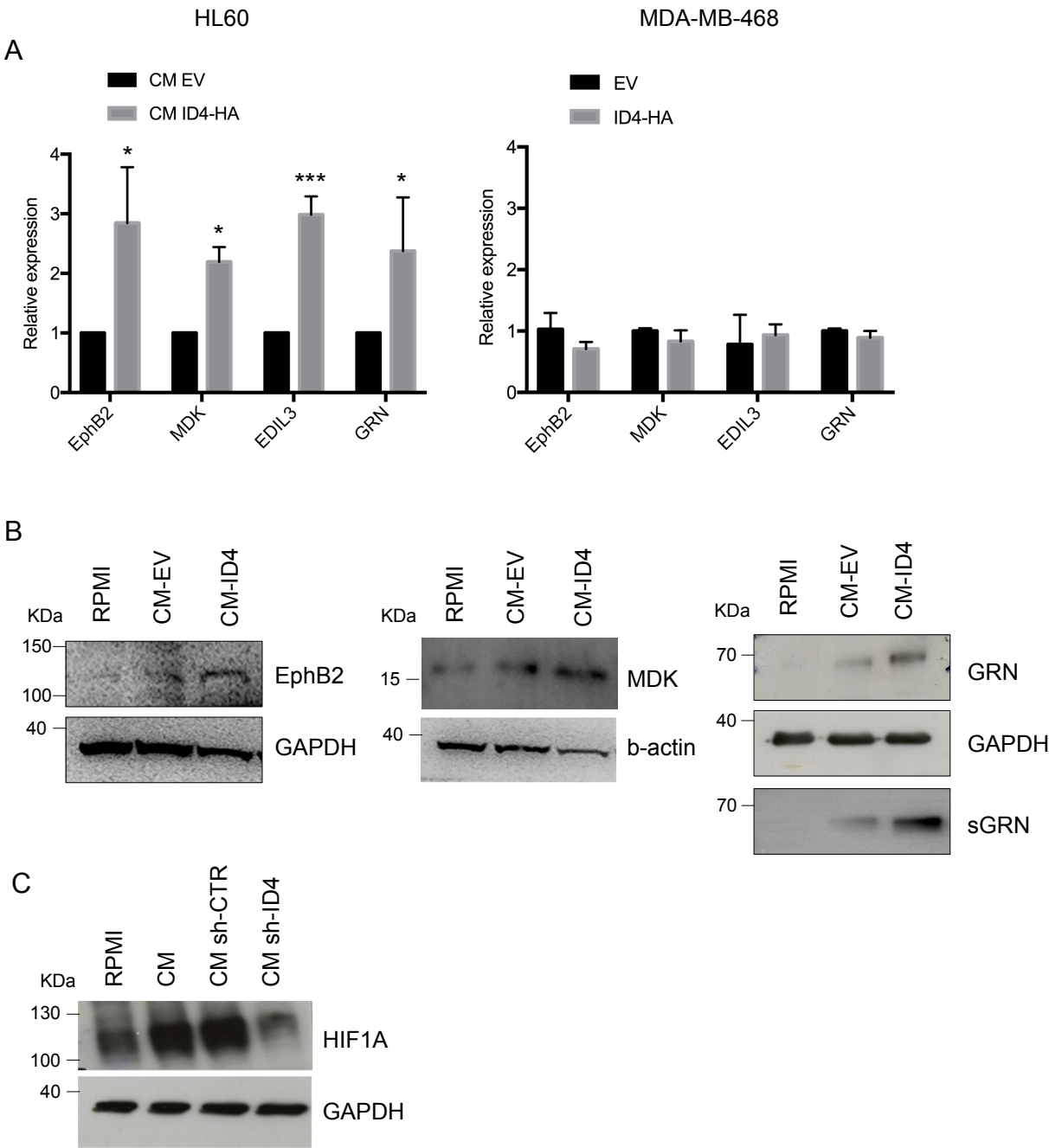

Supplement: Supplementary file 7 — Figure S4 a Modulation of selected genes modulated in the TLDA was validated by RT-qPCR in differentiated HL60 cells cultured in CM from ID4-overexpressing (CM ID4-HA) or control (CM EV) MDA-MB-468 cells (left panel). The same transcripts were analysed in MDA-MB-468 cells transfected with ID4-HA expression vector (ID4-HA) or control empty vector (EV) (right panel). b Expression of EphB2, MDK and GRN protein evaluated by Western blotting on lysates from differentiated HL60 cells cultured as in (a); secreted GRN (sGRN) was evaluated on CM from differentiated HL60 cells in the same conditions. c HIF1A protein expression evaluated by Western blotting in differentiated U937 cells cultured in RPMI medium or in CM from SKBR3 cells stably interfered for ID4 expression (sh-ID4) or control cells (sh-CTR). (PDF 1320 kb) [file 13058_2018_990_MOESM7_ESM.pdf]

Figure S5

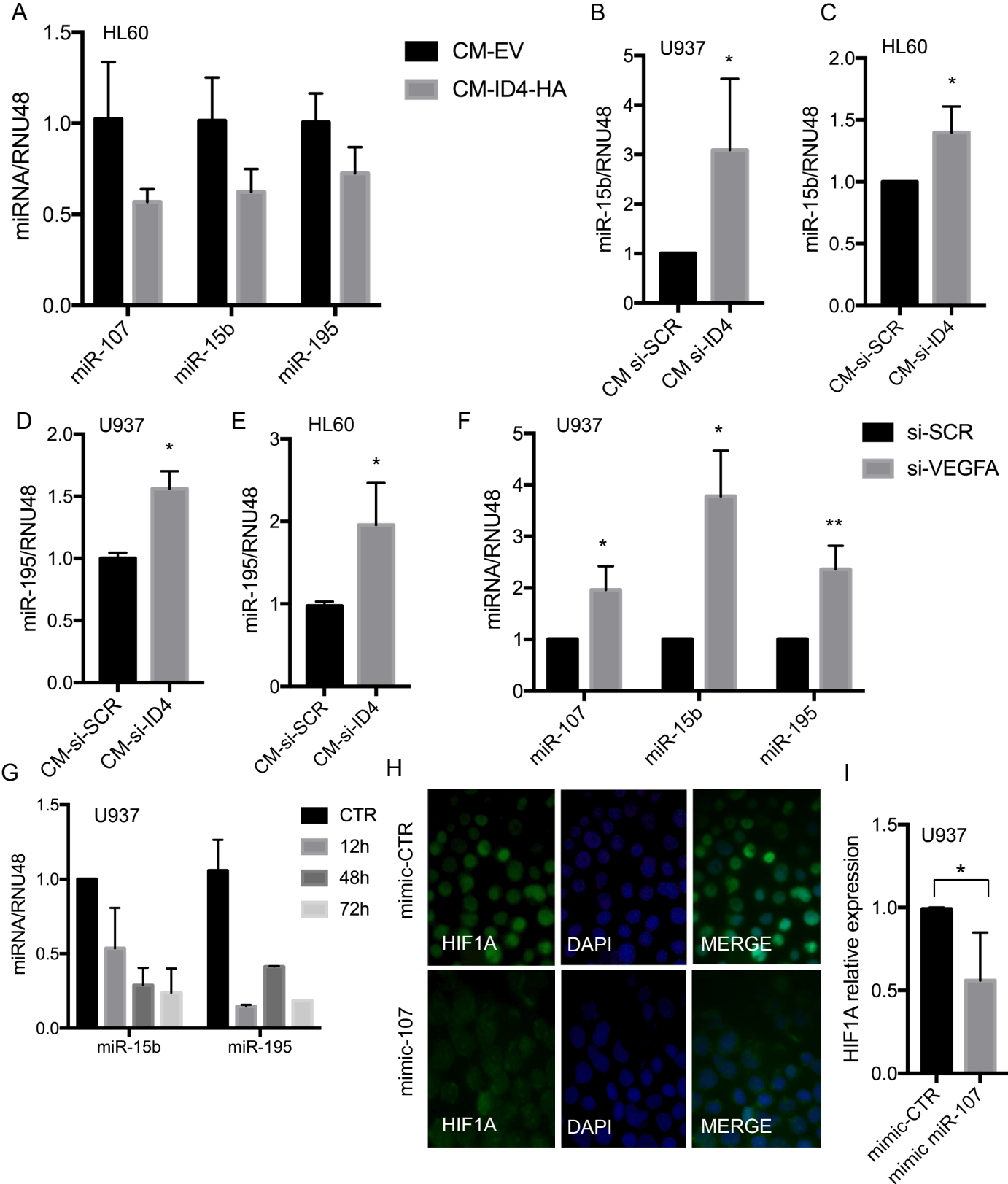

Supplement: Supplementary file 8 — Figure S5 a Expression of miR-107, miR-15b and miR-195 in differentiated HL60 cells cultured with CM from control (CM EV) or ID4-overexpressing (CM ID4) MDA-MB-468 cells. b–e Expression of miR-15b and miR-195 in HL60 and U937 cells cultured with CM from control (si-SCR) or ID4-depleted (si-ID4) BC cells. f miR-107, miR-15b and miR-195 expression evaluated by RT-qPCR in differentiated U937 cells cultured with CM from MDA-MB-468 cells depleted or not of VEGFA expression. VEGFA interference efficiency is shown in Fig. 3i. g Expression levels of miR-15b and miR-195 in differentiated U937 cells cultivated in RPMI medium (CTR) or CM from MDA-MB-468 cells for the indicated time points. h and i HIF1A mRNA (h) and protein (i) expression evaluated, respectively, by RT-qPCR and immunofluorescence in differentiated U937 cells transfected with control mimic or miR-107 mimic and cultured in the presence of CM from MDA-MB-468 cells for 48 hours. (PDF 2150 kb) [file 13058_2018_990_MOESM8_ESM.pdf]

**Figure S6**

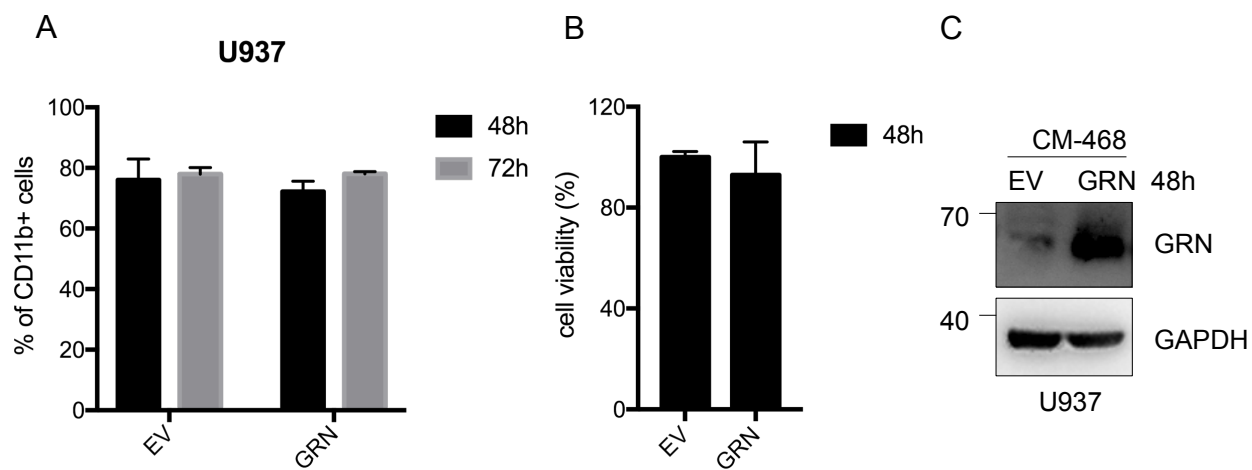

Supplement: Supplementary file 9 — Figure S6 Differentiated U937 cells transfected with an empty vector (EV) or a granulin (GRN) expression vector and subsequently cultivated in the presence of CM from MDA-MB-468 cells were evaluated for their differentiation state (percentage of CD11b+ cells) (a) and for their viability (b) by, respectively, FACS analysis and ATPlite assay at the indicated time points after CM addition. c Overexpression of GRN evaluated by Western blotting. (PDF 141 kb) [file 13058_2018_990_MOESM9_ESM.pdf]
