# Supplementary material for: Expression of ID4 protein in breast cancer cells induces reprogramming of tumour-associated macrophages
Source: Breast Cancer Res. 2018 Jun 19;20:59. doi: 10.1186/s13058-018-0990-2 (PMC6009061; doi:10.1186/s13058-018-0990-2)
Supplement: Supplementary file 2 — Table S1 Characteristics of patients selected for the analysis of ID4 protein expression. (DOCX 17 kb) [file 13058_2018_990_MOESM2_ESM.docx]

**Table S1**. Characteristics of patients selected for the analysis of ID4 protein expression

| 62 TNBC | | |
| --- | --- | --- |
|  | **N** | **%** |
| Histotype |  |  |
| Ductal infiltrating | 58 | 94 |
| other | 4 | 6 |
| Grading |  |  |
| G2 | 20 | 32 |
| G3 | 42 | 68 |
| Lymph node status |  |  |
| N- | 34 | 60 |
| N+ | 23 | 40 |
| Unknown | 5 |  |
| Tumor size |  |  |
| T1 | 33 | 53 |
| T2-T3 | 29 | 47 |
| ID4 |  |  |
| Low (0/1+) | 21 | 34 |
| High (2+/3+) | 41 | 66 |
| CD68 |  |  |
| Low (0/1+) | 16 | 26 |
| High (2+/3+) | 46 | 74 |
| Ki67 |  |  |
| Low (<15%) | 4 | 6 |
| High (>15%) | 58 | 94 |
