# Supplementary material for: Expression of ID4 protein in breast cancer cells induces reprogramming of tumour-associated macrophages
Source: Breast Cancer Res. 2018 Jun 19;20:59. doi: 10.1186/s13058-018-0990-2 (PMC6009061; doi:10.1186/s13058-018-0990-2)
Supplement: Supplementary file 4 — Table S2 Predictive power of ID4, CD68 and the macrophage signature (MacSig) comprising eight widely used markers (CD14, CD105, CD11b, CD68, CD93, CD33, IL4R, CD163) for the mononuclear phagocyte system [37]. Analysis was performed using datasets deposited in the KMplot database [36]. DMFS Distant metastasis-free survival, OS Overall survival. (DOCX 21 kb) [file 13058_2018_990_MOESM4_ESM.docx]

**Table S2**. Predictive power of ID4, CD68 and the macrophage signature (MacSig) comprising eight widely used markers (CD14, CD105, CD11b, CD68, CD93, CD33, IL4R, CD163) for the mononuclear phagocyte system (Murray & Wynn, 2011). Analysis has been performed using datasets deposited in the KMplot database (Gyorffy B et al., 2010).

DMFS: distant metastasis-free survival; OS: overall survival.

| Prognostic indicator | Group | Survival | *P* (Log-Rank) | HR | 95% CI |
| --- | --- | --- | --- | --- | --- |
| ID4 | CD68-high | DMFS | \| <0,0001 \| \| --- \| | \| 5,45 \| \| --- \| | \| 2,48 to 11,96 \| \| --- \| |
|  | CD68-low | DMFS | \| 0,9407 \| \| --- \| | \| 1,029 \| \| --- \| | \| 0,48 to 2,16 \| \| --- \| |
|  | MacSig-high | DMFS | \| 0,0085 \| \| --- \| | \| \| 3,172 \| \| --- \| \| \| --- \| --- \| | \| \| 1,64 to 6,13 \| \| --- \| \| \| --- \| --- \| |
|  | MacSig-low | DMFS | \| 0,1593 \| \| --- \| | \| 1,919 \| \| --- \| | \| 0,77 to 4,75 \| \| --- \| |
|  | CD68-high | OS | \| 0,0416 \| \| --- \| | \| 2,576 \| \| --- \| | \| 1,24 to 5,33 \| \| --- \| |
|  | CD68-low | OS | \| 0,4920 \| \| --- \| | \| 1,44 \| \| --- \| | \| 0,56 to 3,64 \| \| --- \| |
|  | MacSig-high | OS | \| 0,0466 \| \| --- \| | \| 2,748 \| \| --- \| | \| 1,24 to 6,05 \| \| --- \| |
|  | MacSig-low | OS | \| 0,5270 \| \| --- \| | \| 1,355 \| \| --- \| | \| 0,57 to 3,18 \| \| --- \| |
| CD68 | all patients | DMFS | \| 0,9677 \| \| --- \| | \| 0,9893 \| \| --- \| | \| 0,58 to 1,6 \| \| --- \| |
|  | all patients | OS | \| 0,8887 \| \| --- \| | \| 1,036 \| \| --- \| | \| 0,63 to 1,69 \| \| --- \| |
| MacSig | all patients | DMFS | \| 0,3930 \| \| --- \| | \| 0,7915 \| \| --- \| | \| 0,45 to 1,38 \| \| --- \| |
|  | all patients | OS | \| 0,0205 \| \| --- \| | \| \| 0,5636 \| \| --- \| \| \| --- \| --- \| | \| 0,34 to 0,92 \| \| --- \| |
