# Supplementary material for: Expression of ID4 protein in breast cancer cells induces reprogramming of tumour-associated macrophages
Source: Breast Cancer Res. 2018 Jun 19;20:59. doi: 10.1186/s13058-018-0990-2 (PMC6009061; doi:10.1186/s13058-018-0990-2)
Supplement: Supplementary file 5 — Table S3 mRNAs modulated in an ID4-dependent manner in differentiated HL60 cells cultured with conditioned medium from control (CM EV) or ID4-overexpressing (CM ID4) MDA-MB-468 cells. The presence of HIF-1 consensus sequences on promoters was evaluated using the LASAGNA-Search web tool (http://biogrid-lasagna.engr.uconn.edu/lasagna_search/). The presence of putative binding sites for miR-107, miR-15b and miR-195 on 3′-UTR or coding (CDS) sequences of mRNAs was evaluated using the miRWalk analysis tool (http://zmf.umm.uni-heidelberg.de/apps/zmf/mirwalk2/) by selecting the following databases: (1) 3′-UTR analysis = miRWalk, miRanda, miRDB, miRNAMap, Pictar2, RNA22, RNAhybrid, TargetScan; and (2) CDS analysis = miRWalk, miRanda, RNA22, RNAhybrid, TargetScan. (DOCX 22 kb) [file 13058_2018_990_MOESM5_ESM.docx]

|  | Folds vs control (RPMI) | | Two-tailed t-test | | |  |  |  |  |
| --- | --- | --- | --- | --- | --- | --- | --- | --- | --- |
| Gene Symbol | CM EV | CM ID4 | P-value  (CM EV vs RPMI) | P-value  (CM ID4 vs CM EV) | P-value  (CM ID4 vs RPMI) | HIF-1 consensus on promoter | n° of databases predicting miR-107 dependence | n° of databases predicting miR-15b dependence | n° of databases predicting miR-195 dependence |
| ANGPTL4 | 18.96 | 35.87 | 1.09E-07 | 3.74E-02 | 6.40E-06 | Yes | 3(CDS) | - | - |
| ECGF1 | 0.807 | 4.532 | 5.74E-01 | 3.03E-02 | 2.96E-03 | Yes | - | - | - |
| EDIL3 | 1.982 | 5.305 | 9.69E-01 | 1.78E-02 | 1.25E-02 | Yes | 6(3’UTR), 2(CDS) | 2(CDS) | - |
| EPHB2 | 0.889 | 7.339 | 1.18E-01 | 1.62E-03 | 2.62E-05 | Yes | 4(3’UTR), 2(CDS) | 4(3’UTR), 4(CDS) | 5(3’UTR), 4(CDS) |
| FN1 | 0.775 | 4.475 | 8.34E-02 | 1.08E-01 | 2.17E-02 | No | 5(CDS) | 4(CDS) | 5(CDS) |
| GRN | 2.406 | 3.370 | 2.23E-05 | 2.96E-02 | 4.23E-08 | Yes | 3(CDS) | 6(CDS) | 6(CDS) |
| MDK | 2.645 | 3.992 | 8.21E-03 | 4.16E-01 | 1.93E-03 | Yes | - | - |  |
| NRP2 | 1.170 | 2.329 | 4.33E-01 | 7.28E-03 | 4.91E-04 | Yes | 8(3’UTR), 2(CDS) | 9(3’UTR), 6(CDS) | 9(3’UTR), 5(CDS) |
| PRL | 4.563 | 7.031 | 1.04E-05 | 2.58E-02 | 1.92E-10 | Yes | 3(CDS) | 5(CDS) | 4(CDS) |
| VEGFB | 2.039 | 3.703 | 2.63E-03 | 8.36E-02 | 1.63E-04 | Yes | 2(CDS) | 3(3’UTR), 5(CDS) | 4(3’UTR), 5(CDS) |
| VASH1 | 1.035 | 0.470 | 3.22E-01 | 1.00E-02 | 8.93E-02 | Yes | 6(3’UTR) | - | 3(3’UTR) |

**Table S3**.

mRNAs modulated in ID4-dependent manner in differentiated HL60 cells cultured with conditioned medium from control (CM EV) or ID4-overexpressing (CM ID4) MDA-MB-468 cells.

Presence of HIF-1 consensus sequences on promoters was evaluated using the web tool <http://biogrid-lasagna.engr.uconn.edu>.

Presence of putative binding sites for miR-107. miR-15b and miR-195 on 3’-UTR or coding (CDS) sequences of mRNAs was evaluated using the miRWalk analysis tool (http://zmf.umm.uni-heidelberg.de/apps/zmf/mirwalk2/) by selecting the following databases: 1) 3’-UTR analysis= miRWalk, miRanda, miRDB, miRNAMap, Pictar2, RNA22, RNAhybrid, Targetscan; 2) CDS analysis= miRWalk, miRanda, RNA22, RNAhybrid, Targetscan.
